# Deletion of HIF-2α in Dendritic Cells Attenuates Anti-Glomerular Basement Membrane Nephritis

**DOI:** 10.3390/biomedicines13040888

**Published:** 2025-04-07

**Authors:** Jiayi Miao, Junwen Qu, Dawei Li, Ming Zhang

**Affiliations:** Department of Urology, Renji Hospital, School of Medicine, Shanghai Jiao Tong University, Shanghai 200127, China; miaojiayi1999@126.com (J.M.); lidawei3000@126.com (D.L.)

**Keywords:** anti-GBM glomerulonephritis, dendritic cell, hypoxia-inducible factor-2α, p38 mitogen-activated protein kinase

## Abstract

**Background:** Anti-glomerular basement membrane (anti-GBM) nephritis is mediated by autoantibodies and may progress to end-stage renal disease. Although its pathogenesis is not completely understood, dendritic cells (DCs) have been reported to play an important role in this process. Hypoxia-inducible factor-2α (HIF-2α) has been reported to have a regulatory effect on DCs under hypoxic conditions, while no research has investigated its role in autoimmune nephritis. **Methods:** Anti-GBM nephritis was induced in CD11c-specific HIF-2α-deficient and WT mice using nephrotoxic serum (NTS). All mice were divided into four groups: (i) WT+PBS, (ii) CD11c-Cre^+^ Hif2α^fl/fl^+PBS, (iii) WT+NTS and (iv) CD11c-Cre^+^ Hif2α^fl/fl^+NTS. Seven days after induction, renal function, immune cell infiltration and the expression levels of genes in the renal cortex were assessed in each group. **Results:** On day 7, the levels of serum creatinine and blood urea nitrogen and the urine albumin-to-creatinine ratio were lower for mice with DC-specific deletion of HIF-2α compared with their WT counterparts (*p* < 0.05). Histopathological analysis showed that there was less crescent formation in the renal cortex with conditional HIF-2α knockout, and the infiltration of DCs and macrophages was also suppressed (*p* < 0.05). Genes related to antigen processing and presentation were found to be expressed differentially between the two groups, and the activation of the MAPK pathway was affected (*p* < 0.05). Western blot analysis validated that HIF-2α knockout inhibited the phosphorylation of p38 MAPK (*p* < 0.05). **Conclusions:** In this study, we observed a pro-inflammatory effect of HIF-2α in DCs in early anti-GBM nephritis, and the results suggested a regulating effect of HIF-2α on p38 MAPK pathways.

## 1. Introduction

Anti-glomerular basement membrane (anti-GBM) nephritis is a classic autoimmune disease that presents as rapidly progressive glomerulonephritis [1,2]. It is caused by circulating and deposited antibodies against the non-collagenous domain of the α3 chain of type IV collagen which is expressed in kidneys and lungs [3,4]. When anti-GBM nephritis is complicated with pulmonary hemorrhage, the condition is described as “Goodpasture syndrome” and may lead to a fatal outcome [5]. The diagnosis of the disease depends on the findings of anti-GBM antibodies in serum or tissue, and pathological changes from biopsy [1]. Conventional treatment for anti-GBM nephritis includes immunosuppressive therapy and plasma exchange, but even with prompt interference, the prognosis remains poor [6,7].

Dendritic cells (DCs) are major antigen-presenting cells that take up a large portion of leukocytes in the kidney. In anti-GBM nephritis, their role is complicated, and they act as the bridge between innate and adaptive immune responses [8,9,10]. DCs in the mice kidney are characterized by specific surface markers that vary among subsets, and CD11c, a pan-DC marker, is most widely used to identify them. DCs have been reported to activate helper T cells, causing them to produce pro-inflammatory cytokines that can either recruit more effector cells like macrophages or directly promote inflammation [11,12,13]. It has also been reported that the CD103^+^ subset of DCs shows a protective effect in anti-GBM nephritis by recruiting regulatory T cells [14].

Hypoxia-inducible factors (HIFs) are pivotal transcriptional factors that mediate cellular response under the stress of hypoxia and inflammation [15]. Many studies have shown the importance of HIF-1α in regulating cell maturation, migration, and metabolism in DCs, but few have investigated the role of HIF-2α [16,17,18,19]. In our previous studies, we have demonstrated that during ischemia–reperfusion injury, HIF-2α suppresses lipid accumulation and cytokine production in DCs by downregulating CD36 expression, thus ameliorating kidney injury [20,21]. However, the role of HIF-2α in other kidney diseases, such as anti-GBM nephritis, remains uncertain. Given that the activation of HIF-2α is critical for DC functions in aseptic inflammation, we hypothesize that in autoimmune models such as anti-GBM nephritis, HIF-2α may similarly regulate DCs and have an effect on the progression of the disease.

## 2. Materials and Methods

### 2.1. Mice

The conditional knockout murine model with HIF-2α deficiency specifically in CD11c^+^ cells was established through crossbreeding CD11c-Cre mice with Hif2α^fl/fl^ mice, as previously described [20]. Genotypic confirmation was performed using primer sequences provided in Table A1. CD11c-Cre^+^ Hif2α^fl/fl^ and WT male mice between 6 and 10 weeks of age were used in this study. Mice were housed in specific-pathogen-free conditions with a 12 h light/dark cycle and ad libitum access to food and water.

### 2.2. Induction of Anti-GBM Nephritis

All mice were divided into 4 groups: (i) WT+PBS, (ii) CD11c-Cre^+^ Hif2α^fl/fl^+PBS, (iii) WT+NTS and (iv) CD11c-Cre^+^ Hif2α^fl/fl^+NTS. WT mice and transgenic mice were housed together in the same cage one week before the start of the experiment. To prevent intermixing, WT and transgenic mice were ear-tagged and housed in separate cages within the same rack. Cage assignments were verified daily by trained staff. Each mouse was first subcutaneously injected with 1 mg of sheep IgG (Thermofisher, Waltham, MA, USA) dissolved in 0.9% saline and 100 μL of Freund’s complete adjuvant (Thermofisher) 5 days prior to the injection of nephrotoxic serum (NTS) (PTX-001s-Ms; Probetex, San Antonio, TX, USA). On day 0, mice in NTS groups were slowly intravenously injected 10 mL/kg of NTS. Control groups (WT+PBS and CD11c-Cre^+^ Hif2α^fl/fl^+PBS) received PBS instead of NTS but were administered Freund’s complete adjuvant and sheep IgG following the same protocol as NTS groups to ensure identical immunological priming. Weight was measured, and urine samples were collected on days 0, 3 and 7 after injection. All mice were sacrificed on day 7, and blood samples and kidney tissues were collected.

### 2.3. Renal Function Assessment

Commercially available assay kits were used for the quantification of creatinine and blood urea nitrogen (BUN) levels (Jiancheng, Nanjing, China). Urinary albumin was also measured using an assay kit (Bioswamp, Wuhan, China), and the urine albumin-to-creatinine ratio (UACR) was calculated according to the results.

### 2.4. PAS Staining, Immunohistochemistry and Immunofluorescence

Renal tissue specimens were processed for histological evaluation through fixation in 4% paraformaldehyde solution for 24 h followed by paraffin embedding. Histopathological assessment was performed using periodic acid–Schiff (PAS) staining, with quantitative analysis of glomerular pathology based on 50 glomeruli per section for crescent formation evaluation.

For immunohistochemistry staining, the following antibodies were used: anti-CD4 (Servicebio, Wuhan, China), anti-CD11c (Servicebio) and anti-F4/80 (Servicebio). Visualization were developed with diaminobenzidine. Positively stained areas were quantified using ImageJ (version 1.51) with thresholding set to 50–255 intensity and particle size > 50 pixels^2^, and the number of positive cells was counted in a 1 mm^2^ × 1 mm^2^ area in each section.

For fluorescent IgG deposition staining, sections were incubated with Alexa Fluor 488-conjugated Goat Anti-Mouse IgG (Servicebio) for an hour and then mounted.

### 2.5. RNA Sequencing and Data Analysis

RNA isolation from renal cortical tissues was performed using the RNeasy mini kit (Qiagen, Hilden, Germany). Library preparation for transcriptome sequencing was carried out with the TruSeq RNA Sample Preparation Kit (Illumina, San Diego, CA, USA) in accordance with the manufacturer’s protocol. The initial step involved mRNA enrichment through poly-A selection using magnetic beads conjugated with oligo-dT primers. Following library construction, quality control measures were implemented, including quantification using the Qubit 2.0 Fluorometer (Thermo Fisher Scientific, Waltham, MA, USA) and size distribution analysis on the Agilent 2100 bioanalyzer (Agilent Technologies, Santa Clara, CA, USA).

Sequencing preparation involved library normalization to 10 pM concentration, followed by cluster generation on the cBot system. High-throughput sequencing was performed on the NovaSeq6000 platform (Illumina, USA). Processed reads were aligned to the mouse reference genome (GRCm38) using Hisat2 (version 2.0.4) software. Transcript quantification was expressed as FPKM (fragments per kilobase of transcript per million mapped reads). Differential gene expression analysis was conducted with statistical thresholds set at |log_2_FC| > 1 and *p*-value < 0.05. Gene Ontology (GO) functional enrichment, Kyoto Encyclopedia of Genes and Genomes (KEGG) pathway analysis and gene set enrichment analysis (GSEA) were performed using https://www.bioinformatics.com.cn (accessed on 7 November 2024), an online platform for data analysis and visualization.

### 2.6. Real-Time Quantitative PCR

RNA isolation from renal cortical specimens was conducted utilizing TRIzol reagent (Solarbio, Beijing, China). Complementary DNA synthesis was performed using the Hifair AdvanceFast One-step RT-gDNA Digestion SuperMix (Yeasen, Shanghai, China), incorporating simultaneous genomic DNA elimination. Quantitative PCR analysis was carried out on the LightCycler 480II system (Roche, Basel, Switzerland) with Hieff UNICON SYBR Green Master Mix (Yeasen, Shanghai, China). Primers are provided in Table A2. Relative quantification was achieved through normalization to GAPDH expression using the comparative threshold cycle (2^−ΔΔCT^) method.

### 2.7. Western Blot Analysis

Renal cortical tissue samples were homogenized in cold RIPA lysis buffer (Beyotime, Hangzhou, China) supplemented with protease inhibitor cocktail (1:100) using a homogenizer, followed by a 20 min incubation on ice. Protein quantification was performed using the bicinchoninic acid (BCA) method with a commercial kit (Vazyme, Nanjing, China). Electrophoretic separation was conducted on 4–20% gradient Bis-Tris gels, followed by protein transfer onto PVDF membranes. Membranes were blocked using Protein Free Rapid Blocking Buffer (Epizyme, Shanghai, China) for 30 min at room temperature. Following three TBST washes, membranes were incubated with primary antibodies against p38 (14064-1-AP; Proteintech, Rosemont, IL, USA), phospho-p38 (28796-1-AP; Proteintech) and α-tubulin (66031-1-lg; Proteintech) at 4 °C overnight. Subsequent to TBST washing, membranes were exposed to secondary antibodies for 1 h at room temperature. Protein bands were visualized using the Bio-Rad ChemiDoc MP imaging system (Hercules, CA, USA), with semiquantitative analysis performed using ImageJ (version 1.51) software.

### 2.8. Statistical Analysis

Data normality was assessed using the Shapiro–Wilk test, and variance homogeneity via Levene’s test. For multiple comparisons, one-way ANOVA with Tukey’s post hoc test was applied. All statistical computations and graphical representations were generated using GraphPad Prism version 8.0 (San Diego, CA, USA). A probability threshold of *p* < 0.05 was established as the criterion for statistical significance.

## 3. Results

### 3.1. DC-Specific Deletion of HIF-2α in DCs Attenuated Anti-GBM Nephritis

**Figure 1:**
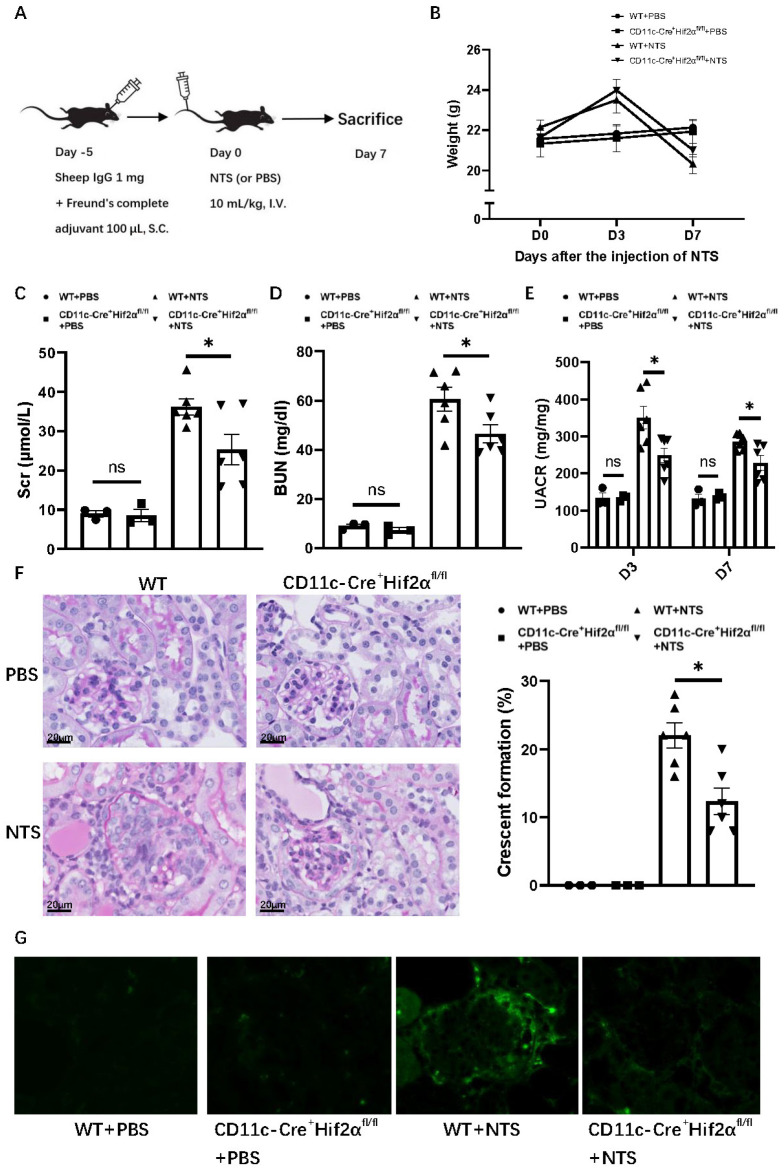
DC-specific HIF-2α deletion attenuated anti-GBM nephritis. (**A**) Experimental schematic of anti-GBM nephritis. (**B**) Weight change of mice after injection of NTS or PBS. (**C**) Serum creatinine and (**D**) BUN level of mice on day 7 after injection of NTS or PBS. (**E**) UACR level of mice on day 3 and day 7 after injection of NTS or PBS. (**F**) Left panel: representative PAS-stained renal sections on day 7 after injection of NTS or PBS (magnification × 400). Right panel: Percentage of glomerulus with crescent formation in PAS-stained renal sections. (**G**) Immunofluorescence images of renal sections on day 7 after injection of NTS or PBS. *n* = 3 per PBS group, *n* = 6 per NTS group. S.C., subcutaneously; I.V., intravenous. All data are mean ± SEM. ns, not significant, * *p* < 0.05. Scale bar = 20 μm.

In order to gain insight into the role of DC HIF-2α in anti-GBM nephritis, we used a murine model with CD11c^+^ cell-specific deletion of HIF-2α that we generated and verified in a previous study [20]. To induce anti-GBM nephritis, each mouse was first subcutaneously injected with 1 mg sheep IgG and then intravenously injected with 10 mL/kg NTS5 days later, while the normal control group was injected with PBS instead of NTS (Figure 1A). The NTS-injected mice showed an increase in weight on day 3, but a decrease on day 7 compared with PBS groups. The transient weight gain on day 3 may reflect edema due to acute kidney injury, while subsequent weight loss correlates with proteinuria and systemic inflammation. Among anti-GBM nephritis groups, WT mice showed a greater weight decline than CD11c-Cre^+^ Hif2α^fl/fl^ mice, but with no statistical significance (Figure 1B). Levels of serum creatinine, BUN and UACR were lower on day 7 after the injection of NTS for CD11c-Cre^+^ Hif2α^fl/fl^ mice, indicating a better retained renal function in the transgene group (Figure 1C–E). Histological analysis of PAS-stained renal sections also showed less severe crescent formation for CD11c-Cre^+^ Hif2α^fl/fl^ mice (Figure 1F). Immunofluorescence images also showed less severe IgG deposition in the CD11c-Cre^+^ Hif2α^fl/fl^ +NTS group than in the WT+NTS group (Figure 1G). Generally, DC-specific HIF-2α deletion leads to a protective effect in early anti-GBM nephritis.

### 3.2. DC-Specific Deletion of HIF-2α Suppressed the Infiltration of DCs and Macrophages in Anti-GBM Nephritis

**Figure 2:**
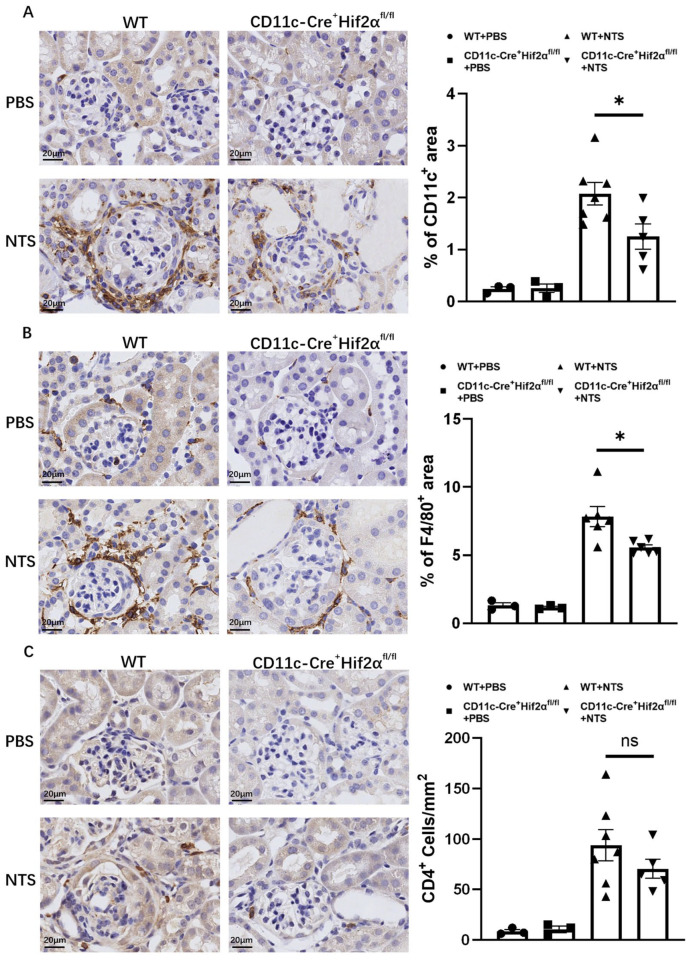
DC-specific HIF-2α deletion inhibited the infiltration of DCs and macrophages, but not CD4^+^ cells, in anti-GBM nephritis. (**A**) Left panel: Representative images of infiltrating CD11c^+^ cells on day 7 after injection of NTS or PBS (magnification × 400). Right panel: Semiquantitative analysis of CD11c^+^ cell infiltration in renal cortex. (**B**) Left panel: Representative images of infiltrating F4/80^+^ cells on day 7 after injection of NTS or PBS (magnification × 400). Right panel: Semiquantitative analysis of F4/80^+^ cell infiltration in the renal cortex (*n* = 3 per PBS group, *n* = 6 per NTS group). (**C**) Left panel: Representative images of infiltrating CD4^+^ cells on day 7 after injection of NTS or PBS (magnification × 400). Right panel: Semiquantitative analysis of CD4^+^ cell infiltration in renal cortex. All data are mean ± SEM. ns, not significant, * *p* < 0.05. Scale bar = 20 μm.

DCs play a major part in the initiation of immune response in early anti-GBM nephritis. In this study, CD11c^+^ cells were stained in renal sections from mice sacrificed 7 days after the injection of NTS or PBS. Significant DC accumulation in the renal cortex was observed, and the percentage of the positively stained area was lower in CD11c-Cre^+^ Hif2α^fl/fl^ mice than in WT mice (Figure 2A). Macrophages are known to have a pathogenic effect in this disease [22,23]. To better assess inflammation, F4/80^+^ cells were also stained, and the result proved that macrophage infiltration in different groups had the same tendency as DC infiltration. In addition, macrophages were more likely to localize around the glomerulus. The pathological difference conformed with the phenotype we found (Figure 2B).

In addition, it is well established that CD4^+^ T-cell activation is an important mechanism in early anti-GBM nephritis and can mediate further immune response in ways like producing cytokines and recruiting downstream inflammatory cells [2,24,25]. In this study, although we observed a decreased number of CD4^+^ cells in CD11c-Cre^+^ Hif2α^fl/fl^ mice in comparison to WT mice, the result showed no significance, suggesting the infiltration of helper T cells was not majorly affected by conditional Hif2α knockout (Figure 2C).

### 3.3. Transcriptomic Analysis of WT Mice Versus CD11c-Cre^+^ Hif2α^fl/fl^ Mice in Anti-GBM Nephritis

To investigate the mechanism underlying different phenotypes between WT and CD11c-Cre^+^ Hif2α^fl/fl^ mice in the anti-GBM nephritis model, transcriptomic analysis was conducted.

**Figure 3:**
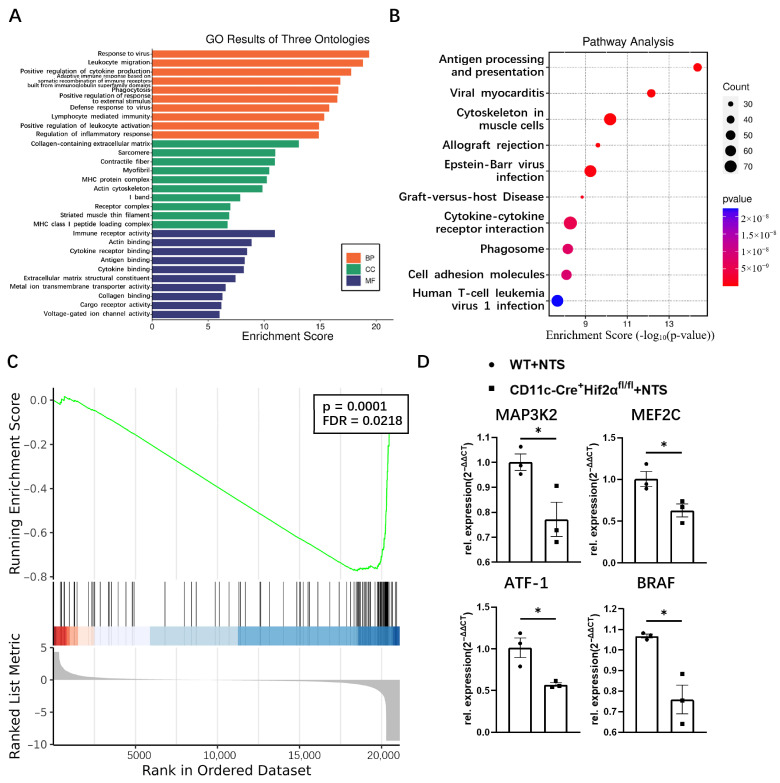
(**A**) GO and (**B**) KEGG enrichment analysis of common differential genes. (**C**) GSEA plot depicting enrichment in FCERI-mediated MAPK activation. (**D**) The mRNA expression levels of MAP3K2, MEF2C, ATF1 and BRAF determined by qPCR, normalized by GADPH, and expressed as 2^−ΔΔCT^ (*n* = 3 per group). All data are mean ± SEM. * *p* < 0.05.

Functional enrichment analysis revealed the attributes of genes in the biological process, cellular component and molecular function categories. The top five enriched GO terms of biological processes were response to virus, leukocyte migration, positive regulation of cytokine production, adaptive immune response based on somatic recombination of immune receptors built from immunoglobulin superfamily domain, and phagocytosis. Immune receptor activity, actin binding, cytokine receptor activity, antigen binding, and cytokine binding were most enriched in the molecular function domain. Most of these terms were related to the regulation of the immune response (Figure 3A). KEGG analysis showed that antigen processing and presentation was the most enriched pathway, indicating dendritic cell function was suppressed in CD11c-Cre^+^ Hif2α^fl/fl^ mice (Figure 3B). GSEA analysis found that the gene set of FcεRI-mediated MAPK activation was greatly affected by the deletion of HIF-2α (Figure 3C). For validation, several representative genes in this pathway, including MAP3K2, MEF2C, ATF-1 and BRAF, were selected for qPCR analysis, and their mRNA expression levels were lower in the CD11c-Cre^+^ Hif2α^fl/fl^ group than in the WT group (Figure 3D). So, we hypothesized that MAPK pathway activation was suppressed with conditional Hif-2α knockout, which led to the attenuation of inflammation.

### 3.4. DC-Specific HIF-2α Deletion Inhibited p38 MAPK Pathway Activation in Anti-GBM Nephritis

**Figure 4:**
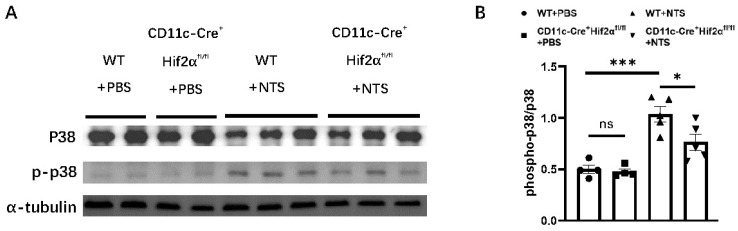
DC-specific HIF-2α deletion inhibited p38 MAPK pathway activation in anti-GBM nephritis. (**A**) Expression of phospho-p38 and p38 in renal cortex on day 7 after injection of NTS or PBS. (**B**) Semiquantitative analysis of phospho-p38-to-p38 ratio (*n* = 4–5 per group). All data are mean ± SEM. ns, not significant, * *p* < 0.05, *** *p* < 0.001.

It has been previously reported that the activation of the p38 MAPK pathway participates in the pathogenesis of anti-GBM nephritis [26,27,28]. Therefore, we investigated the effect of DC-specific HIF-2α deletion on p38 phosphorylation. We found that the phospho-p38-to-p38 ratio was significantly elevated 7 days after the injection of NTS. And this ratio was lower in the CD11c-Cre^+^ Hif2α^fl/fl^ group than in the WT group, indicating a lower level of p38 MAPK activation in the former group (Figure 4).

## 4. Discussion

HIFs are key transcription factors regulating the cellular response to hypoxia and are involved in immune cell regulation in many kidney diseases. So far, no study has investigated the role of HIF-2α in anti-GBM nephritis. In our previous studies, we found a protective effect of HIF-2α upon renal ischemia–reperfusion injury by inhibiting the lipid accumulation of DCs [20]. In anti-GBM nephritis, DCs also play an indispensable role in pathogenesis, although the effect can vary throughout the progression of the disease [11,29].

In this study, HIF-2α showed an aggravating effect in early anti-GBM nephritis, which was different from what we found in an ischemia–reperfusion model. The UACR level of CD11c-Cre^+^ Hif2α^fl/fl^ mice was lower than that of WT mice on day 3 and after, suggesting the participation of HIF-2α in the initial phase of this disease in which kidney injury was predominantly mediated by innate immune response [30]. In anti-GBM nephritis, CD4^+^ T cells are primed by DCs and can then promote inflammation in several ways, including the recruitment of macrophages [12]. Macrophages produce cytokines like tumor necrosis factor and nitric oxide, leading to further damage [8]. We found that the infiltration of DCs and macrophages was less severe in CD11c-Cre^+^ Hif2α^fl/fl^ mice, but we did not confirm the same tendency for CD4^+^ cells. This phenomenon suggested that although DC-specific deletion of HIF-2α did not affect the accumulation of CD4+ T cells, it impeded their macrophage-recruiting capacity by hindering DC-mediated T-cell activation. KEGG analysis showed that antigen process and presentation was the most enriched pathway in this model, and GSEA analysis revealed that genes related to FcεRI-mediated MAPK activation had a significant difference in expression levels between CD11c-Cre^+^ Hif2α^fl/fl^ mice and WT mice. FcεRI is expressed on DCs and is responsible for taking up and processing antigens to induce a secondary T-cell response [31]. Different activation levels of this pathway between the two groups suggested that the T-cell priming function of DCs was affected by HIF-2α deletion. In previous studies, it has been reported that p38 MAPK pathway activation is related to the severity of kidney injury in early anti-GBM nephritis. Studies found an increase in the phosphorylation level of p38 in glomeruli and cortical tubules in the first week after the injection of NTS, and the blockade of p38 was proven to attenuate renal injury [26,27]. In this study, we observed that p38 phosphorylation was suppressed in CD11c-Cre^+^ Hif2α^fl/fl^ mice, suggesting that p38 MAPK pathway inhibition is the downstream effect of HIF-2α knockout.

This study is an exploration of the role of DC-specific HIF-2α in anti-GBM nephritis, but there are still some problems that need to be further resolved. On one hand, in this study, we focused on the early phase of anti-GBM nephritis, while in later phases of this disease, the predominant effector immune cells would differ, and chronic pathological changes like renal fibrosis should also be taken into consideration to better assess renal function. We do not know whether the same difference would appear in a long-term experiment, so in our future studies, we will collect more samples from different time points after the injection of NTS. On the other hand, although our study identified reduced p38 MAPK phosphorylation in mice with DC-specific HIF-2α deletion, the mechanistic link between HIF-2α and p38 MAPK activation remains to be fully elucidated. Future in vitro studies are needed to better confirm this interaction. For example, experiments using p38 MAPK inhibitors or activators in HIF-2α-deficient dendritic cells could further clarify the regulatory hierarchy between HIF-2α and p38 signaling. Since the pathogenesis of anti-GBM nephritis is still not clear, studies involving the effect of DCs or HIF-2α in this model are quite limited. Although we have found the involvement of the p38 MAPK pathway, it is still necessary to investigate cell signaling axes affected by HIF-2α in more detail.

## 5. Conclusions

In summary, our study found that DC-specific deletion of HIF-2α suppressed immune cell infiltration and p38 MAPK activation, thus leading to attenuated kidney injury in early anti-GBM nephritis. This phenomenon has not been previously recognized, so it gives us a better understanding of this autoimmune disease and suggests a new potential therapeutic target for treating rapidly progressive glomerulonephritis.

## Data Availability

The data are presented in the manuscript. Additional information obtained during the experiments is available upon request.

## Abbreviations

The following abbreviations are used in this manuscript:

Anti-GBM	Anti-glomerular basement membrane
DC	Dendritic cell
HIF	Hypoxia-inducible factor
NTS	Nephrotoxic serum
UACR	Urine albumin-to-creatinine ratio
GO	Gene Ontology
KEGG	Kyoto Encyclopedia of Genes and Genomes
GSEA	Gene set enrichment analysis
S.C.	Subcutaneous
I.V.	Intravenous

## Appendix A

**Table A1:** Primer sequences for PCR.

Application	Primers
008407(HIF-2α^fl/fl^ mice)	Forward: GAGAGCAGCTTCTCCTGGAA Reverse: TGTAGGCAAGGA AACCAAGG
008068 (CD11c-Cre mice; transgene)	Forward: ACTTGGCAGCTGTCTCCAAG Reverse: GCGAACATCTTCAGGTTCTG
008068 (CD11c-Cre mice; internal positive control)	Forward: CAAATGTTGCTTGTCTGGTG Reverse: GTCAGTCGAGTGCACAGTTT

**Table A2:** Primer sequences for qPCR.

Application	Primers
MAP3K2 (mouse)	Forward: TTGTCTTTAAGCAGCCCTGAA Reverse: AAAAGTCTTCCGACCGTCATT
MEF2C (mouse)	Forward: AGATCTGACATCCGGTGCAG Reverse: TCTTGTTCAGGTTACCAGGT
ATF-1 (mouse)	Forward: GATTCCCACAAGAGTAACACGAC Reverse: CCTATGCTGTCAGATGAGTCCT
BRAF (mouse)	Forward: CAATTGGCTGGGACACGGACAT Reverse: TTGACAACGGAAACCCTGGAAAAG
